# The Crucial Interplay Between the Lungs, Brain, and Heart to Understand Epilepsy-Linked SUDEP: A Literature Review

**DOI:** 10.3390/brainsci15080809

**Published:** 2025-07-28

**Authors:** Mohd Yaqub Mir, Bilal A. Seh, Shabab Zahra, Adam Legradi

**Affiliations:** 1 Epilepsy Centre, Department of Clinical Sciences, Lund University Hospital, 221 00 Lund, Sweden; 2 Institute of Biochemistry and Biophysics, Polish Academy of Sciences, 02106 Warszawa, Poland; bseh@ibb.waw.pl; 3 Department of Basic and Applied Chemistry, Faculty of Science and Technology, University of Central Punjab, Lahore 54782, Pakistan; shabab.zahra1@hotmail.co; 4 Department of Cell Biology and Molecular Medicine, University of Szeged, H-6720 Szeged, Hungary; legradam@bio.u-szeged.hu

**Keywords:** SUDEP, epilepsy, autonomic nervous system, central apnea, hypoventilation, neurodegeneration

## Abstract

Sudden Unexpected Death in Epilepsy (SUDEP) is a leading cause of mortality among individuals with epilepsy, particularly those with drug-resistant forms. This review explores the complex multisystem mechanisms underpinning SUDEP, integrating recent findings on brain, cardiac, and pulmonary dysfunctions. Background/Objectives: The main objective of this review is to elucidate how seizures disrupt critical physiological systems, especially the brainstem, heart, and lungs, contributing to SUDEP, with emphasis on respiratory control failure and autonomic instability. Methods: The literature from experimental models, clinical observations, neuroimaging studies, and genetic analyses was systematically examined. Results: SUDEP is frequently preceded by generalized tonic–clonic seizures, which trigger central and obstructive apnea, hypoventilation, and cardiac arrhythmias. Brainstem dysfunction, particularly in areas such as the pre-Bötzinger complex and nucleus tractus solitarius, plays a central role. Genetic mutations affecting ion channels (e.g., SCN1A, KCNQ1) and neurotransmitter imbalances (notably serotonin and GABA) exacerbate autonomic dysregulation. Risk is compounded by a prone sleeping position, reduced arousal capacity, and impaired ventilatory responses. Conclusions: SUDEP arises from a cascade of interrelated failures in respiratory and cardiac regulation initiated by seizure activity. The recognition of modifiable risk factors, implementation of monitoring technologies, and targeted therapies such as serotonergic agents may reduce mortality. Multidisciplinary approaches integrating neurology, cardiology, and respiratory medicine are essential for effective prevention strategies.

## 1. Introduction

Sudden Unexpected Death in Epilepsy (SUDEP) refers to the sudden death of a person with epilepsy, not caused by trauma, drowning, or a prolonged seizure. Often, there is evidence of a seizure close to the time of death, but the exact cause remains unclear. SUDEP is one of the main causes of death in people with epilepsy [1], with the risk being up to 20 times higher than in the general population. In one study of childhood-onset epilepsy, 55% of deaths were epilepsy-related, and 30% were due to sudden, unexplained causes—resulting in a 7% risk by age 40 [2]. What makes SUDEP even more challenging is that autopsies often reveal no clear cause [2].

SUDEP can happen at any age but is most common in adults between 20 and 45 years old [3,4]. Higher risks have been observed in patients with uncontrolled epilepsy, those treated at epilepsy centers, people in care facilities, and those who had brain surgery [5]. Being male [5] and having childhood-onset epilepsy may also raise the risk. Socioeconomic factors like poor access to healthcare and medications also contribute [3]. Interestingly, people who only experience absence or myoclonic seizures do not seem to have an increased risk [6]. However, having generalized tonic–clonic seizures (GTCS) significantly increases the risk—by as much as tenfold—especially in people who live alone. Substance or alcohol abuse can also double the risk [7].

Research has identified several specific risk factors. Having more than three GTCS per year can increase SUDEP risk by 10 to 15 times [8]. Other risks include epilepsy that begins early in life, seizures that occur during sleep, and a long history of epilepsy. In children, developmental delay and early-onset epilepsy are additional concerns [9]. Some risks cannot be changed—such as having severe epilepsy or genetic mutations affecting heart-related ion channels like KCNQ1, SCN1A, or SCN5A [10].

A major study known as MORTEMUS found that SUDEP often follows a seizure that leads to brain shutdown (EEG suppression), the cessation of breathing (apnea), and then heart failure [11]. This highlights the critical role of breathing problems in SUDEP, alongside heart and brain function shutdown. The brainstem, which controls breathing, may be suppressed after seizures, leading to dangerous conditions like severe acid buildup, airway spasms, and lack of oxygen [12]. Sleeping in a face-down (prone) position, often seen in SUDEP cases, can worsen these breathing problems [11].

In addition to affecting the brain, epilepsy also puts a strain on the heart. People with generalized or drug-resistant epilepsy are at higher risk for heart issues like irregular heartbeats (arrhythmias), problems with the body’s automatic control system (autonomic imbalance), and structural heart changes [13,14,15,16,17,18]. During a seizure, the nervous system can become unstable, triggering dangerous changes in heart rhythm [11]. Cardiac problems are a major reason why SUDEP occurs and contribute to early death in people with epilepsy [19].

Key mechanisms in SUDEP include the cessation of breathing (central apnea), slow heart rate (bradycardia), complete heart stoppage (asystole), and irregular rhythms, which may happen during or after a seizure. One critical event is postictal generalized EEG suppression (PGES), where the brain temporarily shuts down after a seizure. This affects the medulla oblongata, the brain region that controls breathing and heart rate. When this area fails to work, fatal heart problems can follow [20,21].

Another key factor is autonomic imbalance, where the body’s stress system (sympathetic) becomes overactive, and the calming system (parasympathetic) is suppressed. This weakens the heart over time and increases the risk of deadly arrhythmias [22]. Disruptions in key brain areas like the insular cortex and brainstem also make these irregular heart rhythms more likely, further raising the risk of sudden cardiac arrest [23].

## 2. Materials and Methods

This review was conducted through a comprehensive and integrative analysis of the current literature related to Sudden Unexpected Death in Epilepsy (SUDEP). A systematic search was performed using databases including PubMed, Scopus, and Web of Science to identify peer-reviewed studies published up to May 2025. Keywords were used, such as SUDEP, epilepsy, brainstem dysfunction, cardiac arrhythmia, respiratory failure, central apnea, hypoventilation, autonomic nervous system, and neurotransmitter imbalance. Primary research articles, reviews, meta-analyses, animal studies, and clinical trials were considered.

The selection criteria emphasized high-impact studies that provided mechanistic insights into SUDEP, especially those highlighting interactions among the brain, lungs, and heart. Data from experimental epilepsy models (e.g., pilocarpine, kainic acid, Scn1a^−/−^, Kcna1^−/−^ mice), human neuroimaging studies, post-mortem pathology reports, and electrophysiological recordings were synthesized to map the pathophysiological cascade of SUDEP. The references cited were cross-validated against an external bibliography matrix provided in the Supplementary Materials (Table S1), ensuring accuracy and coverage.

The extracted findings were organized thematically into respiratory, cardiac, and neurological dysfunctions, with additional focus on genetic, molecular, and structural contributors. Emphasis was placed on the translational relevance of preclinical findings to human pathology. Finally, conceptual integration was guided by the triangular framework (brain–lung–heart axis, as shown in the graphical abstract figure), which was used to illustrate the dynamic interplay among the three organ systems implicated in epilepsy-linked SUDEP.

## 3. Results

Based on the extensive literature reviewed and organized in the reference matrix, the following sections explore the key physiological systems implicated in SUDEP: neurological, respiratory, and cardiac. These categories were assigned based on each study’s primary focus, determined through a detailed screening of article titles and abstract content.

**Figure 1:**
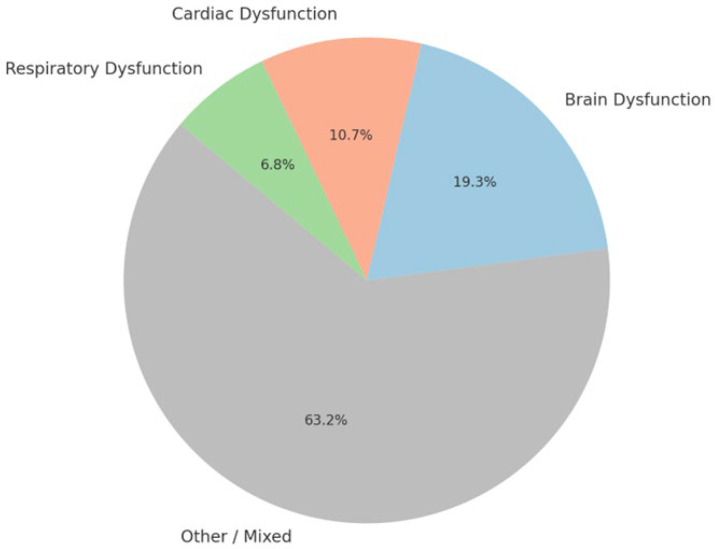
Distribution of literature references by affected system in SUDEP: brain dysfunction (19.3%), cardiac dysfunction (10.7%), respiratory dysfunction (6.8%), and mixed/multisystem mechanisms (63.2%).

To visually represent the scope and focus of the literature included in this review, the Pi chart shown in Figure 1, illustrates the distribution of references across these three domains. As shown, most studies span multiple systems or fall into general or overlapping themes (Other/Mixed). However, a significant portion of the evidence specifically targets brain-related mechanisms (~19%), followed by cardiac (~11%) and respiratory (~7%) dysfunction.

This classification demonstrates that although SUDEP is widely recognized as a multisystem failure, distinct patterns and mechanistic clusters can be extracted from the literature, guiding the structure of the following subsections.

### 3.1. Respiratory Dysfunction in Epilepsy-Related Sudden Deaths

#### 3.1.1. Central Apnea

**Figure 2:**
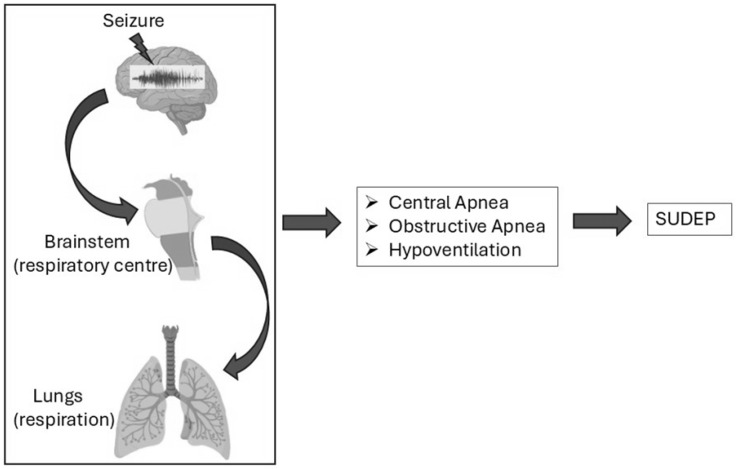
Seizure-induced respiratory dysfunction contributing to SUDEP. Seizures can impair brainstem respiratory centers, affecting lung function and leading to central apnea, obstructive apnea, or hypoventilation—key factors associated with SUDEP risk.

Central apnea, the cessation of breathing due to absent respiratory effort, plays a key role in seizure-related respiratory dysfunction and SUDEP (Sudden Unexpected Death in Epilepsy) [1,2]. Once thought to be primarily cardiac in origin, SUDEP is now linked to central apnea, which often precedes asystole in EMU recordings [11]. It affects nearly half of focal epilepsy patients [12,24], with severe episodes during or after seizures potentially proving fatal [24,25]. These events cause hypoxemia, worsening neurological injury and raising SUDEP risk shown in Figure 2.

Seizures interfere with respiratory centers in the brainstem and autonomic networks [26], notably disrupting the pre-Bötzinger complex and parabrachial nucleus [27], leading to central apnea [28,29]. Temporal lobe epilepsy (TLE) particularly implicates limbic structures in apnea and oxygen desaturation [24,25]. Derera et al. examined the nucleus tractus solitarii (NTS), vital for cardiorespiratory integration [30,31,32], finding GABAergic neuron hyperexcitability in a TLE mouse model due to reduced Kv4 potassium currents [33,34,35,36,37,38]. This hyperexcitability may trigger respiratory and cardiac dysfunction, contributing to SUDEP [28,39,40]. It may also lower the threshold for spreading depolarization, potentially inducing sudden cardiorespiratory collapse [41,42,43].

Brainstem volume loss, especially in the ventrolateral medulla (VLM), including the pre-BötC, has been observed in SUDEP cases [44,45]. Studies report fewer somatostatin and NK1R-expressing neurons and reduced total VLM neurons [46,47,48,49]. Altered serotonergic signaling (lower TPH2 and SERT expression) may reduce 5-HT availability postictally [46]. Although catecholaminergic neuron counts remain unchanged [46,47], altered activation markers and decreased galanin immunolabeling [46,49,50,51,52], along with changes in glial density [53,54,55,56], highlight widespread brainstem pathology linked to SUDEP.

The amygdala is central to this dysfunction; its stimulation triggers apnea, and seizure spread to it often initiates respiratory arrest [57,58,59,60]. It connects directly to the pre-BötC and pontine centers. Nobis et al. found amygdala activation absent in non-apneic seizures, while other rodent studies confirm its control over respiration [61,62,63]. Amygdala and hippocampus stimulation mainly suppresses inspiration, likely by inhibiting brainstem-inspiratory neurons [63].

The insula, involved in both respiratory and cardiac regulation, is also implicated in SUDEP [64]. Insular epilepsy often mimics other focal epilepsies [65], and surgical outcomes improve when it is addressed [66]. Structural insular damage supports its epileptogenic role [67]. Its stimulation causes symptoms such as dyspnea and cardiac disruption, reflecting its autonomic functions and links to SUDEP [66,67,68,69].

Ryvlin et al. described SUDEP as often beginning with a terminal generalized tonic–clonic seizure, followed by tachypnea and then apnea, culminating in respiratory arrest [70]. This pattern mirrors that seen in Kcna1^−/−^ SUDEP models, where failed chemosensory response leads to apnea despite hyperventilation [71,72,73]. Prolonged hypoxia can cause cardiac arrest within minutes [74]. These findings underscore central apnea’s critical role in SUDEP. The amygdala is a promising therapeutic target, and further study of chemosensory instability and brainstem–limbic pathways is essential for SUDEP prevention.

#### 3.1.2. Obstructive Apnea

Unlike central apnea, airway obstruction can significantly worsen respiratory function during and after seizures [75]. Events like seizure-induced laryngospasm—caused by recurrent laryngeal nerve (RLN) hyperactivity—and postictal immobility, especially in the prone position, contribute to airway blockage, leading to hypoxia, hypercapnia, and potential cardiac failure [20,75,76].

Seizures involving the amygdala often cause central apnea, but patients typically remain unaware of their compromised breathing, retaining voluntary respiratory ability when prompted—suggesting a disruption in involuntary drive rather than motor output failure [57,58]. This is supported by studies showing the seizure-induced suppression of medullary serotonergic activity, essential for breathing rhythm [77].

In DBA/2J mice, tracheostomy prevented death by bypassing obstructive apnea caused by laryngospasm. However, some mice still died due to thoracic muscle spasms impairing respiratory effort, revealing two lethal mechanisms: airway obstruction and muscle-induced ventilatory restriction. While ketamine/xylazine reduced thoracic spasms, only eliminating obstruction via tracheostomy ensured survival [78].

Laryngospasm has emerged as a key factor in SUDEP. In animal models and human EEG/EMG studies, persistent inspiratory effort with diminished awareness “respiratory agnosia” has been observed, highlighting the need for comprehensive monitoring in refractory epilepsy [11,79,80].

Obstructive apnea and bradycardia may act synergistically to trigger SUDEP. The MORTEMUS study linked seizure-related RLN hyperactivity to laryngospasm, obstructing the airway and initiating rapid hypoxemia [81]. Only obstructive apnea, not central apnea, was uniquely associated with SUDEP in this model. The intense autonomic response during attempted breathing against a closed airway likely drives cardiopulmonary collapse [81,82].

Experiments confirm that airway occlusion alone, even without seizures, replicates the SUDEP cascade: oxygen desaturation, bradycardia, and cardiac arrest [78]. Central apnea, typically involving minimal autonomic activation, is less dangerous than obstructive apnea, which elicits a strong sympathetic response, making it particularly lethal [39,83,84].

Seizures spreading from the subiculum to the hypothalamic PVN and medulla likely activate both respiratory and autonomic centers, contributing to laryngospasm and apnea [76,85]. In the MORTEMUS study, nine of ten patients showed their steepest heart rate drop just after seizure termination, aligning with late airway obstruction observed in rat models [11,75,86]. In DBA/2J mice, survival with tracheostomy suggests that central apnea is not the primary cause; serotonergic dysfunction and spreading depolarization may act downstream of obstructive apnea [75,87,88,89,90].

These findings point to obstructive apnea—particularly laryngospasm—as a central driver in the SUDEP cascade. The interaction between respiratory obstruction, central neural mechanisms, and cardiac consequences reveals the complexity of SUDEP pathophysiology, emphasizing the critical coordination between the lungs, brain, and heart.

#### 3.1.3. Hyperventilation

Hyperventilation, a process of increased breathing rate and depth beyond what is physiologically necessary, has a long-established association with seizures, even predating the use of EEG [91]. It was historically the first activation method used in EEG to aid in epilepsy diagnosis, activating epileptiform-spiking activity, and less frequently, clinical seizures, in susceptible individuals [91,92], While hyperventilation can trigger seizures in up to 50% of patients with generalized epilepsy, particularly children with absence seizures, it is less effective in focal epilepsy [93]. The mechanisms by which hyperventilation triggers seizures are primarily attributed to hypocapnia, the reduction of carbon dioxide (CO_2_) levels in the blood [94,95]. This leads to respiratory alkalosis, decreased cerebral blood flow, and increased neuronal excitability, making neurons more prone to spontaneous discharges [96,97]. The change in pCO_2_, rather than the absolute level, appears to be the critical factor, with patient-specific sensitivities to hypocapnia. The autonomic nervous system, particularly the sympathetic division, may also play a role [KK]. Studies have shown increased sympathetic responses to hyperventilation in patients with mesial temporal lobe epilepsy, suggesting that sympathetic overactivation may contribute to seizure triggering in some focal epilepsies [98,99,100]. Changes in brain diffusion have also been observed during hyperventilation in patients with temporal lobe epilepsy and hippocampal sclerosis, but not in those without sclerosis or in the controls [101]. Hyperventilation is more effective in activating epileptiform activity in generalized epilepsies, particularly in untreated children with absence epilepsies [102], while less common in focal epilepsies [53], hyperventilation can still be useful in some cases, particularly in medically intractable focal epilepsies [9], and may reflect the pathophysiology of the epileptogenic area [103]. The position of the patient during hyperventilation (supine vs. sitting) may also influence the occurrence of absence seizures [104]. Further research is needed to fully elucidate the complex interplay between hyperventilation, neuronal excitability, autonomic function, and seizure generation in various epilepsy syndromes.

#### 3.1.4. Hypoventilation

Hypoventilation during seizures, marked by reduced breathing rate and depth, impairs CO_2_ clearance and contributes to hypoxemia and hypercapnia, exacerbating seizure effects and increasing SUDEP risk [11,25,105]. It is especially noted during sleep, in the prone position, or alongside pulmonary edema. While congenital central hypoventilation syndrome has been considered, PHOX2B mutations are not commonly found in SUDEP cases [19,106]. Other genes, such as HOXA4 and MeCP2, play critical roles in respiratory control. Disruptions in MeCP2 in various brainstem regions, including the preBötC, affect apnea regulation and hypoxic ventilatory response (HVR) [107,108,109,110,111,112,113,114]. These findings highlight the network complexity involved in breathing regulation.

Animal models show that hypoventilation commonly occurs during ictal and postictal phases [105], possibly due to disrupted respiratory centers or altered baseline breathing [25,115,116]. Studies in epileptic rats and sheep suggest impaired CO_2_ sensing and ventilation–perfusion mismatch as key contributors [117,118,119,120]. Hypoventilation often co-occurs with apnea and is linked to dysfunction in adenosinergic and serotonergic signaling, spreading depolarization, and autonomic dysregulation [121,122,123,124,125,126]. Mouse models (DBA/1, DBA/2) and genetic strains (SCN1A, KCNA1 mutants) demonstrate respiratory arrest and CO_2_ insensitivity during seizures [71,90,122,127,128,129]. Larger models (sheep, baboons) further confirm the role of respiratory failure in SUDEP [120,130].

In Wistar audiogenic rats, increased sympathetic tone, elevated corticosterone, and baroreflex preservation suggest a cardiovascular vulnerability contributing to SUDEP [131,132,133,134,135,136,137]. The TeTX rat model showed a high seizure rate during REM sleep and theta-dominant wakefulness, suggesting a link between sleep states and seizure susceptibility [138,139,140,141,142]. Mechanisms may involve hippocampal excitability and interneuron dysfunction [143,144]. In Scn1aR1407X/+ mice, increased GTCS frequency preceded death, often at night. A ketogenic diet reduced mortality without affecting seizures, suggesting protective effects on brainstem pathways or neuronal structure [70,145,146,147,148,149,150,151].

A baboon study found SUDEP-like pathology—pulmonary edema, myocardial fibrosis, frequent seizures—in those with unexplained deaths, resembling human SUDEP [152,153,154,155,156]. Electrophysiological monitoring is needed to explore potential arrhythmias.

The pilocarpine-induced SE mouse model mimics chronic TLE and SUDEP. Surviving mice show increased glutamatergic excitation of GABAergic NTS neurons, disrupting autonomic regulation and predisposing to cardiorespiratory collapse [25,41,157,158,159,160,161,162,163,164,165]. The kainic acid model highlights seizure-induced laryngospasm and apnea as acute SUDEP mechanisms, though its variability limits long-term applicability [75,166,167].

Hypoventilation may amplify SUDEP risk when combined with arrhythmias [168]. Genetic influences on respiratory vulnerability are under active investigation [169]. Overall, hypoventilation is a key contributor to SUDEP and warrants continued research for targeted prevention.

### 3.2. Brain Dysfunction in Epilepsy-Related Sudden Deaths

**Figure 3:**
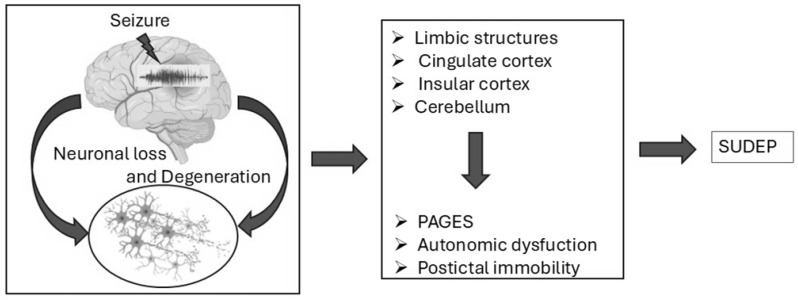
Schematic representation of potential mechanisms leading to SUDEP. Seizures contribute to neuronal loss and degeneration, particularly in regions such as the limbic system, cingulate cortex, insular cortex, and cerebellum. This leads to pathological outcomes, including PAGES, autonomic dysfunction, and postictal immobility, ultimately increasing SUDEP risk.

Brain dysfunction plays a significant role in epilepsy-related sudden deaths and SUDEP. Prolonged and recurrent seizures can lead to neurodegeneration and brain damage over time, impairing cognitive functions, memory, and overall brain health, ultimately influencing an individual’s susceptibility to SUDEP and other complications [170]. This neurodegeneration affects key brain regions involved in autonomic control, directly impacting the interplay between the brain, lungs, and heart—the central theme of SUDEP pathophysiology [170,171] as shown in Figure 3.

#### 3.2.1. Neurotransmitter Imbalances

Neurotransmitters, the chemical messengers of the brain, are fundamental to the regulation of neuronal function. Imbalances in these signaling molecules are critically implicated in the pathophysiology of epilepsy. Specifically, a reduction in gamma-aminobutyric acid (GABA), the principal inhibitory neurotransmitter, coupled with an elevation in glutamate, the primary excitatory neurotransmitter, contributes significantly to seizure activity [172]. These disruptions not only initiate and propagate seizures but also exert profound effects on overall brain health and function. Numerous genetic mutations associated with altered GABA metabolism have been linked to developmental and epileptic encephalopathies (DEEs), with the majority of these mutations observed in genes encoding GABA receptors, such as GABRA, GABRB, and GABRG (encoding GABAA receptors), as well as GABBR (encoding GABAB receptors) [173,174,175]. Mutations affecting any stage of GABA metabolism can disrupt the delicate balance between neuronal excitation and inhibition, predisposing individuals to epilepsy [172]. The dysregulation of GABAergic pathways can result in neuronal hyperexcitability, thereby increasing susceptibility to seizures and the associated autonomic dysfunctions that may contribute to SUDEP. Therapeutic interventions targeting GABAergic signaling aim to restore this crucial balance and mitigate seizure activity [176]. For instance, valproic acid (VPA) exerts its effects by inhibiting GABA degradation and enhancing its synthesis, effectively increasing synaptic GABA levels. Similarly, vigabatrin (VGB), an irreversible inhibitor of GABA transaminase (GABA-T), prevents GABA breakdown, while tiagabine (TGB) blocks GABA transporter 1 (GAT1), reducing GABA reuptake into neurons and glial cells. These mechanisms collectively enhance synaptic GABA concentrations, providing a stabilizing influence on neuronal networks [177]. In epilepsy syndromes linked to GABAA receptor mutations, such as DEEs, enhancing receptor function has demonstrated therapeutic potential. Identifying these specific genetic mutations facilitates the implementation of precision medicine approaches, offering opportunities for targeted treatments [172]. These findings underscore the critical importance of addressing GABAergic dysregulation not only for seizure control but also for its potential influence on autonomic and respiratory regulation, both of which are crucial factors in SUDEP pathophysiology [178,179,180,181]. Addressing neurotransmitter imbalances—particularly, impaired GABAergic signaling—constitutes a vital component of SUDEP research, emphasizing the interconnectedness of brain excitability, respiratory control, and cardiovascular stability. As research continues to elucidate these complex mechanisms, therapeutic strategies tailored to individual neurochemical profiles of epilepsy patients may emerge, potentially reducing SUDEP risk and improving overall disease management.

Glutamate, the predominant excitatory neurotransmitter, and GABA maintain the critical equilibrium between excitation and inhibition in the central nervous system. The disruption of this balance represents a hallmark of epileptic activity and may contribute to SUDEP pathophysiology. Elevations in extracellular glutamate levels have been consistently reported in epileptogenic regions during ictal, peri-ictal, and interictal phases. Microdialysis studies in focal epilepsies have revealed significantly higher glutamate concentrations in epileptogenic regions compared to non-epileptogenic regions or baseline measures, suggesting the region-specific dysregulation of excitatory signaling [182,183,184,185,186]. This excessive glutamate is closely linked to the dysfunction of the glutamate–glutamine cycle. Under normal physiological conditions, glutamate released into the synaptic cleft is efficiently taken up by astrocytes, converted to glutamine by glutamine synthetase, and subsequently recycled back to neurons [187]. However, studies have demonstrated reduced glutamine synthetase expression and activity in epileptogenic hippocampi, resulting in insufficient glutamate clearance and heightened excitotoxicity [184,187]. This dysfunction can exacerbate neuronal hyperexcitability and promote seizure activity, ultimately increasing the risk of seizure-induced mortality, including SUDEP. While glutamate promotes excitation, GABA counteracts this effect through its inhibitory actions [183]. This imbalance between delayed GABA-mediated inhibition and preictal glutamate surges may create a hyperexcitable neuronal milieu, increasing the propensity for prolonged seizures and seizure-related cardiorespiratory dysfunction, both potential precursors to SUDEP [186]. The interplay between glutamate and GABA is further complicated by the presence of mitochondrial dysfunction in epilepsy. Several studies have noted opposing relationships between GABA levels and mitochondrial function in mesial temporal lobe epilepsy and neocortical epilepsy, suggesting differential metabolic regulation between epileptic subtypes [185]. The dysregulation of glutamate and GABA can directly impact neuronal excitability and indirectly influence autonomic and cardiorespiratory control, mechanisms critically implicated in SUDEP. Elevated glutamate levels may precipitate seizures that disrupt brainstem regulatory centers, thus impairing respiration and cardiac function. Similarly, inadequate GABAergic inhibition during seizures could fail to counteract excessive excitation, exacerbating the likelihood of fatal outcomes. Furthermore, glutamate-mediated excitotoxicity and oxidative stress could contribute to neurodegenerative changes that compromise vital autonomic functions over time [184,188]. Recent studies have also implicated alterations in glutamate transporter expression and function in SUDEP, further highlighting the importance of glutamate homeostasis in this condition [189]

Abnormalities in serotonin (5-HT) neurotransmission have been increasingly implicated in SUDEP pathogenesis. This association arises from serotonin’s crucial role in modulating respiratory and arousal functions, both of which are integral to brainstem activity and are demonstrably disrupted in SUDEP. Evidence supporting the involvement of 5-HT in SUDEP, while largely circumstantial, is nonetheless compelling [173,190]. 5-HT neurotransmission participates in seizure suppression, with studies demonstrating that 5-HT decreases seizure frequency and severity in both animal models and human epilepsy patients [190]. Many commonly prescribed antiepileptic drugs are known to elevate extracellular 5-HT concentrations, which may partially explain their therapeutic efficacy [191,192,193]. Reduced 5-HT levels observed post-seizure in animal models further suggest that serotonin dysfunction may contribute to postictal respiratory suppression [194]. Animal studies provide further evidence for the protective role of serotonin against seizure-induced respiratory arrest. Pretreatment with selective serotonin reuptake inhibitors (SSRIs) has been shown to prevent seizure-related respiratory arrest in DBA mice, a well-established model of SUDEP [88,195]. Similarly, mice deficient in 5-HT2C receptors exhibit a heightened incidence of audiogenic seizures and are prone to respiratory arrest following seizures [196]. These findings underscore the importance of 5-HT signaling in maintaining respiratory stability during and after seizures. In human studies, the administration of SSRIs has been associated with reduced oxygen desaturation during seizures, a recognized biomarker for SUDEP risk [196]. This observation highlights the clinical relevance of enhancing 5-HT activity to mitigate SUDEP risk. The parallels drawn between SUDEP and sudden infant death syndrome (SIDS), which is also linked to brainstem 5-HT system abnormalities, further strengthen the hypothesis of serotonin involvement in SUDEP pathogenesis [64,197]. Although direct evidence definitively linking 5-HT dysfunction to human SUDEP remains limited, the convergence of animal and clinical data strongly suggests that serotonin plays a critical role in regulating the complex interplay between seizures, respiratory function, and arousal. This positions the 5-HT system as a crucial focus for both understanding and mitigating SUDEP. Serotonin (5-HT) dysfunction has been implicated not only in SUDEP but also in SIDS, highlighting overlapping mechanisms related to arousal and respiratory failure [AA]. Infants who succumb to SIDS often exhibit defects in the 5-HT system, including reduced binding of 5-HT1A receptor ligands in the raphe nuclei, a brainstem region vital for the serotonergic regulation of respiration and arousal [198]. Furthermore, an increased number of immature 5-HT neurons and decreased 5-HT levels in the medulla have been observed in SIDS victims, suggesting developmental delays in serotonergic maturation [197,199]. A key similarity between SIDS and SUDEP lies in the context of the fatal events: both conditions involve impaired arousal and respiratory responses during states of central nervous system depression—sleep in SIDS and the postictal state in SUDEP. In SIDS, these deficits are attributed to the delayed maturation of 5-HT neurons and diminished neuronal firing during sleep [196]. In SUDEP, the transient dysfunction of 5-HT neurons is hypothesized to occur due to seizure activity propagating into the brainstem, thereby disrupting the serotonergic modulation of respiratory and arousal mechanisms. Recent research has investigated the role of specific 5-HT receptor subtypes, such as 5-HT1A and 5-HT2A receptors, in SUDEP [196,200], suggesting that these receptors may be promising therapeutic targets. Importantly, preventive measures in SIDS, such as the “Back to Sleep” campaign advocating supine sleeping positions, have significantly reduced its incidence, further implicating positional factors in the role of 5-HT dysfunction [196]. Similarly, in SUDEP, targeting serotonergic dysfunction through interventions such as SSRIs has shown promise in mitigating postictal respiratory depression and seizure-induced death in animal models [88,195]. These parallels underscore the crucial role of 5-HT in regulating responses to external stressors during vulnerable states, thus reinforcing the need to explore serotonergic pathways for therapeutic intervention in SUDEP. In summary, the dysregulation of these key neurotransmitter systems disrupts the intricate communication pathways within the brain, directly impacting respiratory and cardiac control and contributing to the lethal cascade observed in SUDEP. This emphasizes the crucial interplay between the brain, lungs, and heart in this complex condition.

#### 3.2.2. Neurodegeneration and Brain Damage

Structural and functional alterations, particularly in the brainstem, hippocampus, amygdala, and insular cortex, have been linked to increased SUDEP risk. Brainstem atrophy, especially when extending into the midbrain, impairs autonomic control, a critical factor in SUDEP [170,201]. Seizure spread to the amygdala may contribute to respiratory depression via its functional connection with the medullary respiratory network [57], directly impacting lung function. Structural changes, such as increased gray matter volume in the right anterior hippocampus/amygdala and parahippocampus, and decreased volume in the posterior thalamus, further suggest compromised oxygen regulation in individuals at higher SUDEP risk, again highlighting the brain–lung connection [202,203]. Intrinsic or acquired insular damage in refractory epilepsy patients is also a potential risk factor, as insular dysfunction correlates with autonomic nervous system abnormalities and peri-ictal respiratory or cardiac impairments, contributing to SUDEP [204,205]. Neuroimaging studies in temporal lobe epilepsy (TLE) patients have further identified altered functional connectivity in brain regions involved in autonomic, respiratory, and cardiac regulation, potentially serving as biomarkers for SUDEP risk [170,206].

Specifically, patients at high risk for SUDEP exhibit reduced connectivity in a subnetwork encompassing the thalamus, brainstem, anterior cingulate cortex, putamen, and amygdala, and increased connectivity in regions like the medial/orbital frontal cortex, insula, limbic areas, subcallosal cortex, brainstem, thalamus, caudate, and putamen [206]. The posterior thalamus, crucial for oxygen sensing and breathing (the lung–brain connection), demonstrates disrupted links with the brainstem in high-risk patients, potentially contributing to respiratory failure [207]. Reduced thalamic–cingulate connectivity further implicates disruptions in cardiorespiratory and blood pressure regulation (linking the brain, heart, and lungs), which could lead to prolonged hypotension, a possible SUDEP mechanism [208]. The putamen, integral to autonomic and motor regulation, shows reduced connectivity with the cingulate cortex, impairing critical ANS communication [209]. Similarly, diminished connectivity between the amygdala and brainstem in high-risk patients may result in sustained apnea or failure to recover from seizure-induced hypoventilation [170,210].

Several other factors contribute to this complex interplay. The prone position exacerbates respiratory compromise, as impaired arousal and compromised brainstem autoresuscitation mechanisms prevent patients from clearing airway obstructions caused by bedding [211]. Serotonin dysfunction has been implicated in SUDEP, with defective serotonin-producing neurons in epilepsy patients reducing ventilatory responses to rising CO_2_ levels and compromising arousal mechanisms, leading to fatal outcomes during airway obstruction [62,77,212]. Endogenous adenosine, which increases during seizures, provides anticonvulsant effects but also inhibits cardiac and respiratory functions. Prolonged seizures combined with impaired adenosine clearance may trigger excessive adenosine release, contributing to SUDEP [123,213,214,215,216]. Similarly, seizures may activate endogenous opioids, producing central hypoventilation and postictal apnea, which heighten SUDEP risk [217].

Seizure spread to the amygdala has been specifically linked to respiratory arrest due to disruption of the medullary respiratory network, leading to a loss of spontaneous breathing and awareness of dyspnea, further emphasizing the brain’s pivotal role in SUDEP pathophysiology [57]. Ion channel gene mutations, including those in sodium (e.g., SCN1A, SCN8A) and potassium channels (e.g., KCNA1, KCNQ2), disrupt autonomic control and postictal recovery, increasing SUDEP risk [218,219]. Central hypoventilation and apnea may also result from the seizure-induced release of endogenous opioids, with polymorphisms in the ARRB2 gene amplifying the desensitization of brainstem opioid receptors, leading to severe postictal apnea and higher SUDEP susceptibility [217]. Genetic variants affecting glutamatergic and GABAergic neurotransmission can also disrupt the excitatory–inhibitory balance, influencing seizure severity and centrally mediated autonomic dysfunction, thereby increasing the risk of SUDEP [217]. These findings highlight the genetic underpinnings of autonomic dysfunction and their pivotal role in seizure-related respiratory compromise. Structural neuroimaging studies have revealed reduced posterior thalamic gray matter and increased right hippocampal and amygdala volumes in high-risk SUDEP patients [220]. Significant volume loss in the dorsal mesencephalon and damage to central autonomic control regions, such as the limbic system, disrupt critical autonomic and respiratory regulation [221,222]. Seizure-related autonomic dysfunction, including impaired cardiac and respiratory control, is consistently linked to SUDEP risk [223].

Functional connectivity (FC) studies in TLE patients at high risk of SUDEP reveal significant disruptions in brain networks involved in autonomic and respiratory regulation. Reduced FC has been identified in key regions, including the thalamus, brainstem, ACC, bilateral putamen, and left amygdala, all of which play critical roles in regulating breathing, cardiac function, and blood pressure [206,207]. The posterior thalamus, essential for oxygen sensing and relaying respiratory signals, exhibits disrupted connections with the brainstem, aligning with volumetric studies showing reduced gray matter in the thalamus of high-risk SUDEP patients [224]. The putamen shows diminished connectivity with the ACC, potentially impairing communication between motor and autonomic pathways [225]. Similarly, reduced connectivity between the amygdala and brainstem is concerning, given the amygdala’s influence on respiratory nuclei and its association with terminal apnea in SUDEP cases [58,210]. Conversely, high-risk SUDEP patients also show enhanced FC, primarily involving connections between the medial/orbital frontal cortex, insula, and limbic regions (amygdala and hippocampus). These changes could represent compensatory or maladaptive adaptations in autonomic regulation pathways [206].

Temporal lobe epilepsy (TLE), with seizure foci in temporal lobe structures such as the hippocampus, amygdala, entorhinal cortex, and subiculum [226,227], is characterized by significant neurodegeneration and brain damage. Hippocampal neurodegeneration, a hallmark of TLE, leads to structural and functional impairments [228]. Neurodegenerative changes in TLE include aberrant mossy fiber sprouting [229], granule cell dispersion, and astrogliosis [230], all of which contribute to altered neural circuitry and seizure propagation. The neurodegenerative processes in epilepsy are intricately linked to SUDEP. Recurrent seizures and associated excitotoxicity drive neuronal loss, metabolic dysfunction, and progressive structural damage, particularly in the hippocampus [231]. This damage is often exacerbated by ictal and interictal activity, contributing to oxidative stress and inflammatory responses [231]. In TLE, higher seizure frequency and prolonged seizure duration correlate with severe hippocampal atrophy, which impairs the brain’s ability to regulate critical autonomic functions, including respiration and cardiac activity [232]. TLE patients are particularly vulnerable to cognitive deficits, with the nature and severity of these deficits depending on the location and extent of underlying neuropathology [233,234]. In TLE, hippocampal sclerosis (HS) is a common neuropathological feature associated with widespread cognitive impairments, including verbal and visual memory deficits, language difficulties, and postictal psychosis, particularly when both hemispheres are affected [235,236]. Ictal and interictal activities further exacerbate these cognitive disturbances by contributing to excitotoxic damage, inflammatory responses, and disruptions in neural network integrity.

This brain damage plays a pivotal role in the pathophysiology of SUDEP, as the hippocampus and other affected regions are involved in the central regulation of breathing and cardiovascular functions. Dysregulation in these systems due to neurodegeneration can result in impaired responses to seizure-induced apnea or cardiac arrhythmias, which are critical events in SUDEP. Thus, understanding the mechanisms of neurodegeneration in epilepsy provides essential insights into the vulnerability of the brain to fatal outcomes such as SUDEP.

#### 3.2.3. Genetic Mutations and Aberrant Neurogenesis

Mutations in the Kv1.1 (Kcna1) subunit of voltage-gated potassium channels cause significant brain damage and neurodegeneration in epilepsy, with direct implications for SUDEP [233]. In humans, these mutations are associated with temporal lobe epilepsy (TLE) and episodic ataxia type I [233]. Mouse models recapitulate these phenotypes, exhibiting aberrant postnatal neurogenesis in the dentate gyrus, contributing to hippocampal enlargement—an early hallmark of epileptogenesis [237]. This aberrant neurogenesis stems from intrinsic defects in progenitor cell depolarization and extrinsic excitatory inputs, such as NMDA receptor activation and dysregulated GABA signaling, accelerating cellular proliferation [238]. This hyperactive neurogenesis integrates immature neurons into hippocampal circuits, promoting aberrant synchronization and recurrent seizures, destabilizing network excitability, and potentially driving further neurodegeneration and elevating SUDEP risk [235].

System x-c (Sxc), a sodium-independent cystine–glutamate antiporter, a heterodimeric complex composed of xCT and 4F2 chains, primarily located on astrocytes and possibly other glial cells and neurons [239], is implicated in this process. Upregulated during epileptogenesis, Sxc modulates extracellular glutamate, linking excitotoxicity, inflammation, and oxidative stress to neurodegeneration [234]. In a Kcna1 knockout mouse model, genetic deletion of Sxc decreased aberrant neurogenesis in the dentate gyrus, preventing hippocampal enlargement despite persistent severe epilepsy [240]. Increased xCT protein expression has been observed in resected hippocampi from patients with mesial temporal lobe epilepsy [241] and xCT-deficient mice, who have shown increased chemoconvulsant seizure thresholds. This role is further supported by studies investigating the effects of Sxc manipulation on epileptogenesis. For instance, xCT deletion prolongs the latency to the first spontaneous seizure and reduces the number of spontaneous seizures after self-sustained status epilepticus (SSSE). Similarly, in the corneal kindling model, xCT deletion decreases the number of focal to bilateral tonic–clonic seizures (FBTCS) and lowers mean seizure scores, although these effects are modest [242]. In the pentylenetetrazol (PTZ) kindling model, xCT deletion reduced the percentage of mice that became fully kindled [243]. Furthermore, pharmacological inhibition of Sxc with sulfasalazine (SAS) reduced seizures in mice that had undergone pilocarpine-induced SE, but not in control mice, suggesting that Sxc plays a role in modulating the seizure circuit resulting from epileptogenesis [244]. These findings suggest that Sxc contributes to the long-term changes associated with epilepsy development and progression, rather than simply influencing acute seizure activity, while xCT deletion reduced microglial and astrocytic activation after SSSE [244].

#### 3.2.4. Role of Adenosine and Purinergic Signaling

Mesial temporal lobe epilepsy (MTLE) is characterized by significant brain damage, including irreversible biochemical and structural changes in the hippocampus and neocortical regions, contributing to epilepsy pathophysiology and increased SUDEP risk. The dysregulation of extracellular ATP and adenosine is a critical factor in epilepsy progression and neurodegeneration. High-frequency neuronal firing during seizures increases ATP and adenosine levels, which act on adenosine receptors to modulate neuronal excitability [245]. The adenosine A2A receptor (A2AR), expressed in the hippocampus and other brain regions, plays a crucial role in controlling synaptic transmission and plasticity [246]. A2AR activation increases glutamate release and impairs glutamate uptake, leading to an imbalance in excitatory synaptic transmission that may contribute to the hyperexcitability seen in epilepsy [246]. A2AR activation in astrocytes and microglia is also important for modulating neuronal network activity during intense or prolonged seizures. Astrocytes, through A2AR signaling, can influence synaptic glutamate levels and are involved in memory formation [206]. In epilepsy models, including kainate-induced seizures, A2AR levels are upregulated in astrocytes, suggesting a role in the maladaptive neuroplasticity and neurodegeneration associated with epilepsy [247]. These findings indicate that A2AR, by modulating glutamate dynamics, contributes to the progressive neuronal damage and dysfunction seen in MTLE, which may also increase SUDEP susceptibility [248]. Neurodegeneration in epilepsy, particularly MTLE, is thus driven by a combination of altered purine signaling, astrocyte and microglial activation, and glutamate dysregulation. The upregulation of A2AR in both neurons and glial cells contributes to network hyperexcitability and sustained neuronal damage, central to the development of drug-resistant epilepsy and a heightened risk of SUDEP [206,246,247]

In sclerotic TLE, characterized by tonic–clonic convulsions causing progressive brain damage and exacerbating seizures [249], the adenosine modulation system plays a significant role [250]. Increased neuronal activity, especially during seizures, elevates extracellular adenosine levels. While the A1 adenosine receptor (A1R) system is generally considered neuroprotective, reducing seizures and protecting against neuronal damage [251], repeated seizures reduce A1R density and function, limiting its effectiveness [252,253]. This loss of A1R function contributes to progressive brain damage in epilepsy [254]. Conversely, A2AR is upregulated in sclerotic brain regions in epilepsy models and TLE patients [255]. A2AR activation increases glutamate release, enhances NMDA receptor activity, and promotes neuroinflammation [247,256,257]. These effects contribute to pathological changes in the hippocampus during epileptic episodes. While A2AR antagonism has shown neuroprotective effects in various brain conditions [246,258], the exact role in seizure-induced neurodegeneration remains uncertain [259,260]. The dysregulation of adenosine receptors, particularly A1R and A2AR, contributes to chronic brain damage and increased SUDEP risk.

#### 3.2.5. Mossy Fiber Sprouting, Dynorphin, and CDKL5 Deficiency

Aberrant mossy fiber sprouting, where mossy fibers project into the inner molecular layer, is observed in TLE. This sprouting, originating from the dentate gyrus and associated with hilar mossy cell loss, is thought to exacerbate epileptic activity [228], contributing to seizure perpetuation and neuronal damage. The perturbation of glutamatergic signaling, particularly through AMPA receptors and mossy fiber sprouting, plays a critical role in excitotoxic damage and neurodegeneration in epilepsy and may contribute to SUDEP [228,261,262,263].

Dynorphin A (1–17), an opioid peptide normally found in the hippocampal mossy fiber system, exhibits altered distribution in TLE [262]. In TLE specimens, Dyn-IR structures appear in the molecular and granule cell layers, showing distinct distribution patterns. The extent of these aberrant Dyn-IR structures correlates with the degree of cell loss in the polymorph and CA3 regions, as well as granule cell dispersion [264]. This sprouting of mossy fibers and their axon collaterals suggests the formation of new, potentially excitatory circuits. These reorganized fibers, containing dynorphin, could contribute to recurrent excitatory circuits that facilitate synchronous firing and epileptiform activity [265]. This aligns with observations in experimental epilepsy models, where mossy fiber sprouting is implicated in seizure propagation and brain damage, potentially contributing to SUDEP [266].

Studies using Emx1- and CamK2α-derived Cdkl5 conditional knockout (cKO) hemizygous male mice have revealed recurrent spontaneous seizures and aberrant mossy fiber sprouting in the hippocampus of Emx1-derived Cdkl5 cKO mice [266,267]. Increased frequencies of spontaneous and miniature excitatory postsynaptic currents in dentate granule cells of the Emx1-cKO mice further support the epileptic phenotype, suggesting that hyperexcitability in glutamatergic neurons plays a role in the seizures observed in CDKL5 deficiency disorder (CDD) [16,268]. Thus, CDKL5 disruption in glutamatergic neurons not only leads to spontaneous seizures but also enhances excitatory signaling, potentially fostering neurodegeneration and exacerbating SUDEP risk.

These diverse mechanisms—genetic mutations, aberrant neurogenesis, altered purinergic signaling, mossy fiber sprouting, and CDKL5 deficiency—converge on a common pathway: neurodegeneration and disruption of neuronal networks crucial for autonomic control. This disruption directly impacts the delicate balance between the brain, lungs, and heart, making individuals with epilepsy, particularly those with TLE and related conditions, more vulnerable to SUDEP. The interplay of these factors underscores the complexity of SUDEP pathophysiology and highlights the need for further research to develop targeted therapies.

### 3.3. Cardiac Dysfunction in Epilepsy-Related Sudden Deaths

**Figure 4:**
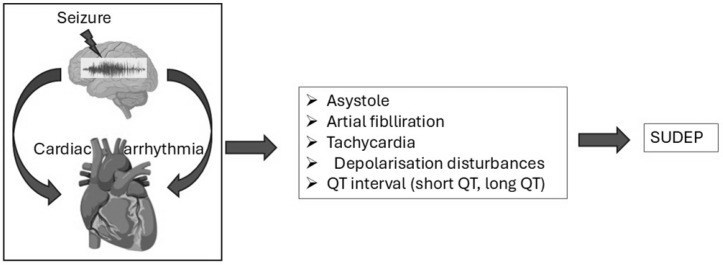
Seizure-induced cardiac arrhythmias and their role in SUDEP. Seizures can trigger cardiac dysfunction, including asystole, atrial fibrillation, tachycardia, and QT interval abnormalities, all of which may contribute to the risk of Sudden Unexpected Death in Epilepsy.

Seizures, particularly generalized tonic–clonic seizures, can significantly disrupt autonomic regulation, leading to cardiac arrhythmias such as tachycardia, bradycardia, or asystole [20,269]. The insular cortex and brainstem, critical regions for autonomic control, are frequently impacted during seizures. The insular cortex manages signals related to heart rate and blood pressure, while the brainstem coordinates vital autonomic reflexes. When seizures affect these areas, they cause imbalances between sympathetic and parasympathetic activity. Overactivation of the sympathetic system results in rapid heart rates (tachycardia), whereas parasympathetic overstimulation can lead to dangerously slow heart rates (bradycardia) or temporary cardiac arrest (asystole), all of which substantially increase the risk of cardiac arrest during or following seizures [52]. As depicted in Figure 4, seizures disrupt autonomic regulation, provoking arrhythmias such as asystole, atrial fibrillation, and QT interval abnormalities, which are key contributors to SUDEP.

Seizures also cause an increase in catecholamines, including adrenaline and noradrenaline, which can temporarily impair heart function in a condition termed “neurogenic stunned myocardium”. This transient myocardial dysfunction arises due to the excessive catecholamine release during the seizures. The resulting calcium overload in cardiac myocytes reduces contractility and predisposes the heart to ventricular arrhythmias. Patients experiencing frequent or drug-resistant seizures are particularly susceptible to this phenomenon and have an increased risk of SUDEP [270]. These effects are further aggravated by genetic predispositions such as mutations in ion channel genes (e.g., SCN5A, KCNQ1), which compromise the electrical stability of cardiac cells, thereby increasing the risk of arrhythmias [17,19]. Genetic factors also play a critical role in associating epilepsy with cardiac risk. Mutations in genes like SCN1A are commonly associated with Dravet syndrome, which disrupt neuronal and cardiac excitability and increase the likelihood of prolonged seizures and autonomic instability. This instability may lead to bradycardia and other life-threatening arrhythmias [271,272]. Similarly, KCNA1 mutations, which impair potassium ion flow, can be provoked early after-depolarizations and arrhythmias, which affect both epilepsy and non-epilepsy populations. These insights emphasize the need for routine cardiac monitoring in epilepsy patients, particularly those at elevated risk. Tools like continuous electrocardiogram (ECG) monitoring, wearable seizure detection devices, and heart rate variability (HRV) analysis provide valuable information about autonomic dysfunction. Early interventions, such as beta-blockers to manage sympathetic overactivity or antiepileptic drugs to reduce seizure frequency, can reduce arrhythmia risks and improve overall outcomes [273,274].

#### 3.3.1. Autonomic Nervous System Dysregulation and SUDEP Risk

The ANS plays an important role in the connection between seizures and SUDEP. Seizures that affect critical areas such as the insular cortex and brainstem disrupt the delicate balance between the sympathetic and parasympathetic branches of the ANS. This imbalance results in irregular heart rhythms, including tachycardia and bradycardia, which substantially elevate the risk of SUDEP [11,21]. A key factor contributing to this autonomic instability during seizures is impaired baroreceptor reflex sensitivity. This dysfunction amplifies sympathetic overactivity while reducing parasympathetic recovery, which makes individuals more prone to arrhythmias. Baroreflex gain analysis during interictal and postictal phases holds promise as a tool for identifying patients at higher risk of autonomic instability and SUDEP [275]. Excessive sympathetic activation during seizures can lead to elevated heart rates, vasoconstriction, and increased blood pressure, which place significant strain on the cardiovascular system. While such responses are typically adaptive under normal conditions, their increased activation during seizures results in chaotic heart rhythms that increase the risk of arrhythmias and cardiac arrest [19,273]. Conversely, parasympathetic overactivation during the seizure, particularly those that originate in the temporal lobe, can cause a dramatic slowing of the heart, occasionally leading to asystole. These abrupt and extreme shifts in the autonomic output increase the chances of fatal outcomes [23,275].

#### 3.3.2. Genetic Contributions to Autonomic Dysregulation

Genetic factors significantly intensify the relationship between epilepsy, ANS dysregulation, and cardiac risks. Mutations in ion channel genes such as SCN1A and KCNA1 can interfere with the excitability of both neurons and cardiac cells. SCN1A mutations, often associated with Dravet syndrome, disrupt sodium channel function and lead to prolonged seizures and autonomic instability, which increase the risk of bradycardia and other arrhythmias [271]. Similarly, KCNA1 mutations affect potassium channels and can compromise the heart’s electrical stability and raise the likelihood of early after-depolarizations and arrhythmias. The release of stress hormones such as epinephrine and norepinephrine during seizures amplifies these autonomic imbalances. The increase in these hormones increases the risk of arrhythmias, particularly in individuals with drug-resistant epilepsy and frequent seizures [22,274,276]. The combination of hormonal fluctuations and genetic predisposition creates a state of increased susceptibility to SUDEP.

#### 3.3.3. Monitoring and Biomarkers of ANS Dysregulation

Understanding the role of the ANS in SUDEP has highlighted the importance of monitoring autonomic function. Tools like heart HRV analysis provide insights into ANS stability. A decrease in HRV during and after seizures signals impaired autonomic regulation and is associated with an elevated risk of SUDEP. Low HRV reflects increased sympathetic activity or inadequate parasympathetic recovery, both of which are critical contributors to cardiac dysfunction in epilepsy patients [277,278]. For high-risk patients, monitoring autonomic biomarkers such as HRV and catecholamine levels can serve as early indicators of developing cardiac dysfunction. This can facilitate timely interventions, including beta blocker therapy to control sympathetic overactivity and targeted antiepileptic treatments to reduce the seizure frequency. Such personalized strategies may help lower the risk of SUDEP and improve overall outcomes for epilepsy patients [273].

#### 3.3.4. Postictal Cardiac Dysfunction and the Role of Continuous Monitoring

##### Postictal Phase and Cardiac Health

The postictal phase, the period immediately following a seizure, is an important time for assessing cardiac health. During this phase, autonomic function is often severely compromised, which leads to cardiac complications such as prolonged QT intervals, bradyarrhythmias, and other arrhythmogenic conditions that contribute to an elevated risk of SUDEP [279,280]. These issues arise from persistent sympathetic overactivation or parasympathetic withdrawal, both of which destabilize cardiac rhythms and predispose patients to fatal arrhythmias.

**Figure 5:**
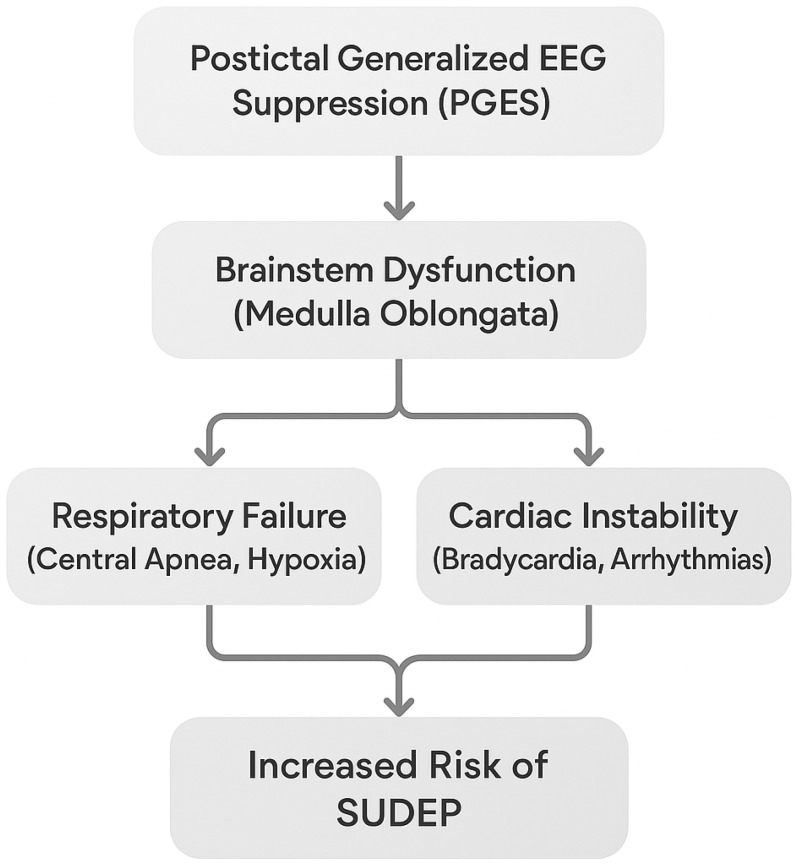
Pathway from PGES to SUDEP via brainstem dysfunction, respiratory failure, and cardiac instability.

Postictal generalized EEG suppression (PGES), characterized by reduced brain activity after seizures, is closely associated with brainstem dysfunction. Figure 5 illustrates the mechanistic relationship between PGES, brainstem dysfunction, and SUDEP. PGES leads to impaired autonomic control via brainstem dysfunction, which then contributes to both respiratory failure and cardiac instability, ultimately increasing the risk of SUDEP.

PGES-induced brainstem dysfunction affects both respiratory and cardiac functions, increasing SUDEP risk. Monitoring postictal cardiac and respiratory markers may aid in identifying high-risk individuals. The medulla oblongata, which controls important cardiorespiratory functions and becomes impaired during PGES, leads to bradycardia, asystole, or central apnea. These mechanisms play a significant role in SUDEP by disrupting heart rhythm during the vulnerable postictal phase [20,21]. Additionally, patients experiencing severe PGES mostly exhibit electrocardiographic markers of cardiac instability, such as QT dispersion and T-wave alternans. These markers provide valuable insight into cardiac vulnerability. Monitoring such parameters during the postictal phase may enable the early identification of high-risk individuals [281].

##### Importance of Postictal Monitoring

The real-time monitoring of cardiac function during the postictal phase provides important insights into seizure-related cardiac abnormalities. Ambulatory electrocardiogram (ECG) devices enable the detection of arrhythmic events such as heart rate variability disturbances, bradyarrhythmias, or QT prolongation, which might not be apparent during routine clinical evaluations [39,282]. The early detection of these disruptions lowers the risk of SUDEP and enables timely treatments. It provides details about heart dysfunction by combining postictal ECG data with advanced autonomic evaluations like HRV analysis. The balance between sympathetic and parasympathetic activity is examined by HRV analysis, and it is linked to the risk of SUDEP and the severity of seizures [283].

##### Emerging Role of Wearable Monitoring Devices

The management of cardiac risks associated with epilepsy is being transformed by wearable technologies, such as adhesive chest sensors and wrist-worn ECG monitors. Continuous cardiac monitoring in real-time environments is made possible by these technologies, allowing for the early diagnosis of autonomic changes or seizure-induced arrhythmias [284,285]. Data collected by these devices can be transmitted in real-time to healthcare providers, facilitating prompt intervention when abnormalities are identified [286,287]. Recent advancements have enabled the development of multi-parameter systems that integrate cardiac, respiratory, and neurological data to provide a comprehensive view of patient health [288]. These systems are particularly beneficial for identifying subtle postictal changes that may otherwise go unnoticed [289]. The integration of wearable devices into routine epilepsy management has the potential to significantly enhance long-term outcomes for patients and reduce the risk of SUDEP [290]. The incorporation of machine learning algorithms into wearable technology has further improved their capacity to detect cardiac risks associated with seizures [291]. These systems can analyze multi-parameter data, such as heart rate variability and motion patterns, to predict seizure onset and autonomic instability [292]. Devices such as Empatica’s Embrace2 have shown effectiveness in clinical assessments, offering real-time notifications to carers and healthcare professionals, thereby facilitating a prompt response [286,293].

#### 3.3.5. Mechanisms Linking Respiratory and Cardiac Dysfunction in SUDEP

##### Overlap Between Respiratory and Cardiac Dysfunction

Respiratory failure is recognized as an important factor in SUDEP, although its interplay with cardiac dysfunction causes a dangerous overlap that markedly increases risk during and after seizures [293]. Respiratory abnormalities resulting from seizures, including central apnea, hypoventilation, and obstructive airway events, lead to hypoxia and hypercapnia, imposing significant strain on the cardiovascular system [294,295,296]. These alterations aggravate arrhythmogenic conditions by increasing sympathetic activity and suppressing parasympathetic recovery, hence elevating the chance of fatal cardiac consequences [297]. Central apnea, frequently triggered by brainstem impairment during convulsions, significantly affects cardiorespiratory stability [11]. The medulla oblongata, responsible for regulating respiration and heart rate, becomes compromised, resulting in concurrent respiratory arrest and bradyarrhythmias [20]. This dual dysfunction is a characteristic mechanism in SUDEP, as hypoxia resulting from apnea might increase the likelihood of fatal arrhythmias [298,299]. Obstructive and central apneas during seizures lead to severe hypoxemia, which increases sympathetic overactivation and raises arrhythmogenic risk [300]. Myocardial stress from hypoxemia diminishes oxygen supply to heart tissue, elevating the risk of fatal ventricular arrhythmias [23,301]. These findings emphasize the significance of continuous monitoring of respiratory and cardiac functions to enhance understanding of SUDEP pathogenesis. Ongoing monitoring of these characteristics may facilitate the identification of high-risk episodes and suggest preventive measures [302].

##### Neurotransmitter Release and Autonomic Imbalance

The excessive release of neurotransmitters like serotonin, glutamate, and catecholamines during seizures significantly disrupts the balance between the respiratory and cardiac systems [303]. Serotonin, for instance, influences respiratory drive and autonomic tone, and its dysregulation during seizures may lead to both apnea and arrhythmias [87]. Glutamate-induced excitotoxicity disrupts brainstem function, impairing central respiratory control and triggering sympathetic overdrive [304,305]. Catecholamine surges, which frequently occur during generalized seizures, increase myocardial stress by increasing heart rate and blood pressure. This increased sympathetic activity raises the likelihood of arrhythmias and simultaneously increases oxygen demand, resulting in a detrimental cycle of cardiorespiratory instability [290,298].

##### Importance of Multimodal Monitoring

The intricate relationship between respiratory and cardiac dysfunction emphasizes the need for integrated monitoring strategies in epilepsy management. Multimodal systems that evaluate respiratory parameters, including oxygen saturation and end-tidal CO_2_ levels, along with ECG and HRV, offer a complete picture of the patient’s cardiorespiratory health [306]. These devices allow clinicians to identify apnea-induced arrhythmias and intervene effectively to avoid negative consequences [158,307,308]. The integration of wearable and portable monitoring devices into clinical practice provides an effective way of obtaining real-time data. Devices that monitor respiratory and cardiac parameters are essential for identifying at-risk individuals in healthcare settings. These tools enhance the accuracy of SUDEP risk classification and provide personalized management strategies to improve patient safety through continuous observation [11].

## 4. Discussion

Sudden Unexpected Death in Epilepsy (SUDEP) is a devastating outcome for individuals with epilepsy, especially those with uncontrolled generalized tonic–clonic seizures (GTCS). The phenomenon is now widely acknowledged to result from a complex interaction between the brain, heart, and lungs, with failures in one or more of these systems contributing to sudden fatality. Emerging evidence strongly suggests that respiratory compromise, autonomic imbalance, and seizure propagation into critical brainstem centers form a lethal cascade culminating in SUDEP.

Among the most critical events observed in SUDEP is central apnea, characterized by a cessation of respiratory effort due to neural suppression. Monitoring data from epilepsy units have shown that central apnea often precedes cardiac arrest, suggesting that it may be an initiating event rather than a secondary consequence of seizure activity [11,24]. Brainstem structures such as the pre-Bötzinger complex and the nucleus tractus solitarius (NTS) play pivotal roles in maintaining respiratory rhythm. Seizure propagation into these areas can suppress their function, thereby triggering central apnea [25,27]. Specifically, the hyperexcitability of GABAergic neurons in the NTS has been demonstrated in pilocarpine-induced epilepsy models, which is directly linked to impaired cardiorespiratory regulation and increased SUDEP vulnerability [33,38]. This hyperexcitability also reduces the threshold for spreading depolarization, a pathological neuronal event that can lead to acute respiratory and cardiac shutdown [41].

In addition to central mechanisms, obstructive apnea, particularly resulting from laryngospasm, is increasingly recognized as a major factor in SUDEP. Seizure-induced laryngospasm leads to intense inspiratory efforts against a closed airway, resulting in progressive hypoxemia and autonomic overload. Animal models have provided compelling evidence of this mechanism: Stewart et al. identified high-frequency EMG signatures of laryngospasm in both EEG and EKG recordings during seizures, closely correlating with SUDEP-like outcomes [78]. Further supporting this, human cases also show signs of obstructive apnea with absent arousal responses during the postictal period [79,80]. Importantly, in models where tracheostomy bypasses the upper airway, survival increases significantly, confirming that mechanical airway occlusion is often the immediate cause of death [81].

Cardiac dysfunction also plays a crucial role. Seizures induce profound shifts in autonomic balance, with sympathetic overactivation or vagal dominance capable of inducing bradycardia, asystole, or ventricular arrhythmias [20,77]. Genetic mutations, particularly in SCN1A and KCNA1, compromise ion channel function in both neurons and cardiomyocytes. These mutations are seen in high-risk epilepsy syndromes such as Dravet syndrome and lead to reduced cardiac excitability, increasing the likelihood of arrhythmias during or after seizures [70,71].

A unique focus has been placed on the amygdala, a limbic structure that interfaces directly with brainstem respiratory centers. Electrical stimulation of the amygdala in both animal and human studies has been shown to induce central apnea, with patients sometimes unaware of the breathing arrest, a condition referred to as respiratory agnosia [57,58,59]. These findings support the hypothesis that seizure spread to the amygdala can silence involuntary respiratory drive without affecting voluntary motor output, greatly complicating postictal recovery.

Another area of growing interest is the serotonergic system, particularly the role of 5-HT in maintaining postictal respiration. In mouse models, pretreatment with SSRIs significantly reduces the incidence of seizure-induced respiratory arrest [87,88]. Human studies show reduced oxygen desaturation during seizures in patients taking SSRIs, suggesting a protective effect [195]. This parallels observations in sudden infant death syndrome (SIDS), which is also associated with impaired serotonergic function in the medulla, a region crucial for arousal and respiratory control [197,198,199].

Moreover, the integration of these dysfunctions creates a vulnerable scenario, especially in high-risk contexts like sleep, the prone position, or when patients are unattended. The transition from a seizure to a fatal event often involves a combination of impaired arousal, autonomic instability, and compromised respiratory control, all occurring within a critical timeframe where intervention is difficult.

## 5. Conclusions

The overall understanding is that SUDEP is not the result of a single system failure but rather a cascading interplay between central apnea, obstructive airway events, and cardiac dysregulation, each potentially exacerbated by seizure-induced neural suppression or genetic susceptibility. Interventions aimed at reducing seizure spread to brainstem structures, enhancing serotonergic tone, and mitigating airway obstruction hold promise in reducing SUDEP risk. Continued research into neural cardiorespiratory circuits, genetic biomarkers, and pharmacological modulation is essential to develop effective, personalized preventive strategies.

## 6. Limitations and Future Research Directions

Despite the comprehensive scope of this review, several limitations must be acknowledged. First, while the integration of findings from human studies and animal models provides valuable insights into SUDEP mechanisms, translational gaps remain. Many animal models, such as those involving pilocarpine or kainic acid-induced seizures, do not fully replicate the chronic and heterogeneous nature of human epilepsy, particularly in terms of seizure semiology, comorbidities, and environmental influences.

Second, the reliance on retrospective data from epilepsy monitoring units (EMUs) and postmortem analyses introduces potential biases, including underreporting of peri-ictal respiratory and cardiac events and variability in diagnostic criteria for SUDEP. The absence of standardized protocols for autonomic and respiratory monitoring across centers further complicates the interpretation of findings.

Third, while genetic studies have identified several candidate mutations associated with SUDEP, including those affecting ion channels and neurotransmitter systems, the functional consequences of many variants remain poorly understood. Moreover, gene–environment interactions and epigenetic modifications are underexplored areas that may significantly influence SUDEP susceptibility.

Future research should prioritize the development of chronic, multimodal animal models that better mimic the human epileptic condition, including spontaneous seizures, comorbid autonomic dysfunction, and sleep-related vulnerabilities. Longitudinal studies incorporating wearable biosensors and real-time cardiorespiratory monitoring in high-risk patients could provide critical data on premonitory signs and facilitate early intervention strategies.

Additionally, the role of neuroinflammation, glial dysfunction, and metabolic alterations in SUDEP pathogenesis warrants deeper investigation. Advanced neuroimaging techniques and single-cell transcriptomics may uncover novel biomarkers and therapeutic targets. Finally, interdisciplinary collaboration among neurologists, cardiologists, geneticists, and bioengineers is essential to translate mechanistic insights into effective, personalized prevention strategies.

## Supplementary Materials

The following supporting information can be downloaded at: https://www.mdpi.com/article/10.3390/brainsci15080809/s1, Table S1. Classification of studies included in the review based on study type. References are divided into human and animal studies, encompassing experimental, clinical, and observational work. This categorization was used to support the synthesis of evidence on SUDEP mechanisms across model systems. Each reference number corresponds to the full citation in the main reference list. (References [3,5,6,7,9,11,20,24,25,27,30,33,36,37,38,41,42,43,44,46,50,51,52,53,57,63,64,65,66,67,69,70,71,74,75,76,77,78,80,81,85,87,91,93,101,102,103,104,107,110,111,113,117,118,119,120,126,135,138,139,150,151,152,153,157,159,162,163,167,168,169,170,172,183,185,190,194,195,196,198,206,207,208,209,211,217,220,223,224,225,228,229,230,231,232,233,234,235,237,238,239,240,241,243,244,246,247,248,249,250,251,254,270,271,273,275,281,283,288,289,290,291,292,293,297,300,302,303,306,307] are cited in the Supplementary Materials).

## Abbreviations

SUDEP	Sudden Unexpected Death in Epilepsy
EEG	Electroencephalography
GTCS	Generalized Tonic–Clonic Seizures
ANS	Autonomic Nervous System
PGES	Postictal Generalized EEG Suppression
RLN	Recurrent Laryngeal Nerve
HVR	Hypoxic Ventilatory Response
TLE	Temporal Lobe Epilepsy
DEE	Developmental and Epileptic Encephalopathy
GABA	Gamma-Aminobutyric Acid
VPA	Valproic Acid
VGB	Vigabatrin
TGB	Tiagabine
SSRI	Selective Serotonin Reuptake Inhibitor
SIDS	Sudden Infant Death Syndrome
5-HT	Serotonin (5-Hydroxytryptamine)
Sxc	System x-c (Cystine-Glutamate Antiporter)
SAS	Sulfasalazine
A1R	Adenosine A1 Receptor
A2AR	Adenosine A2A Receptor
MTLE	Mesial Temporal Lobe Epilepsy
SE	Status Epilepticus
FBCTS	Focal to Bilateral Tonic–Clonic Seizures
CDKL5	Cyclin-Dependent Kinase-Like 5
KD	Ketogenic Diet
HRV	Heart Rate Variability
