# Supplementary material for: The Crucial Interplay Between the Lungs, Brain, and Heart to Understand Epilepsy-Linked SUDEP: A Literature Review
Source: Brain Sci. 2025 Jul 28;15(8):809. doi: 10.3390/brainsci15080809 (PMC12384494; doi:10.3390/brainsci15080809)
Supplement: Supplementary file 1 [file brainsci-15-00809-s001.zip › brainsci-3698409-supplementary.pdf]

Table S1. Classification of studies included in the review based on study type. References are divided into human and animal studies, encompassing experimental, clinical, and observational work. This categorization was used to support the synthesis of evidence on SUDEP mechanisms across model systems. Each reference number corresponds to the full citation in the main reference list.

Study Type	Reference Numbers
Human	[3,5–7,9,11,20,24,25,53,57,64–67,69,75–78,80,81,86,92,94,102–105,151,152,169,170,173,191,195–197,199,207–210,212,218,221,224–226,229–233,235,240–242,244,245,250–252,255,271,272,274,276,282,284,289–293,307,308]
Animal	[11,20,27,30,33,36–38,41–44,46,50–52,63,70,71,74,78,81,88,108,111,112,114,118–121,127,136,139,140,153,154,158,160,163,164,168,171,184,186,207,229,234,236,238,239,247–249,294,298,301,303,304]
